# Juxta-papillary duodenal diverticula are associated with pyogenic liver abscesses: a case control study

**DOI:** 10.1186/s12876-022-02120-4

**Published:** 2022-02-07

**Authors:** Uday Shankar, Priyanka Bhandari, Ankur Panchal, David Weeks, Helen Wu, Fufei Chen, Narinder Maheshwari, Raghav Bansal, Aaron Walfish, Joel Baum, Priya A. Jamidar, Joshua Aron

**Affiliations:** 1 Gastroenterology Division, Department of Medicine, Icahn School of Medicine at Elmhurst Hospital, Elmhurst, NY 11373 USA; 2 Digestive Disease Center, Trinity Health of New England and St. Mary’s Hospital, 133 Scovill Street, Suite 101, Waterbury, CT 06706 USA; 3 Department of Medicine, Icahn School of Medicine at Elmhurst Hospital, Elmhurst, NY 11373 USA; 4 Department of Radiology, Icahn School of Medicine at Elmhurst Hospital, Elmhurst, NY 11373 USA; 5 Connecticut Convergence Institute, Department of Psychiatry, University of Connecticut Health Center, Farmington, CT 06312 USA; 6 Connecticut Convergence Institute, University of Connecticut Health Center, Farmington, CT 06312 USA; 7 Department of Medicine, University of Connecticut Health Center, Farmington, CT USA; 8 Division of Digestive Diseases and Advanced Endoscopy, Yale University School of Medicine, New Haven, CT USA

**Keywords:** Pyogenic liver abscess, Cryptogenic liver abscess, Juxta-papillary duodenal diverticulum

## Abstract

**Background:**

Juxta-papillary duodenal diverticulum (JPDD) has been associated with obstructive jaundice and ascending cholangitis. Potential mechanisms include periampullary colonization of pathogenic bacteria and mechanical obstruction. However, the relation of JPDD with pyogenic liver abscess (PLA) has not been reported. Moreover, approximately one third of patients with PLA have no identifiable risk factors and are labelled as “cryptogenic”. We hypothesized that JPDD is an unidentified risk factor for cryptogenic PLA and the aim of this study was to examine this association.

**Methods:**

We conducted a retrospective chart review to identify cases of PLA (n = 66) and compare those to matched controls (n = 66). 66 patients met the study inclusion criteria of a diagnosis of PLA using computerized tomography (CT) imaging and either positive culture or confirmed resolution after antibiotic therapy. Patients with diagnoses of amebic liver abscess, traumatic liver abscess, post cholecystectomy liver abscess, concurrent acute cholecystitis, and hepatobiliary malignancy were excluded. Controls were identified from a radiology database and matched one-to-one with the cases by age and sex. Demographic and clinical data was extracted from electronic medical records. CT scan images of all cases and controls were reviewed by a single expert radiologist to identify the presence of JPDD. Statistical tests including Chi-square and t-test with multiple logistic regression were used to examine the group differences in JPDD and other factors.

**Results:**

Among 132 study samples, 13.6% (9/66) of the cases were found to have JPDD, compared to 3.0% (2/66) among controls (*p* = 0.03). This corresponded to an odds ratio (OR) of 5.05 [OR 5.05; CI 1.05–24.4] on multiple logistic regression analysis. In addition, 1/3rd of PLA cases with JPDD had no other traditional risk factors (cryptogenic PLA). However, a statistically significant association of JPDD with cryptogenic PLA could not be established possibly because of a small number of cases. We found significantly high rate of diabetes mellitus (DM) (42.4%; n = 28/66) among cases compared to controls (21.2%; n = 14/66; *p* = 0.01).

**Conclusion:**

We found a significant association between JPDD and PLA. We need studies with larger sample sizes to confirm this relationship and to explore if JPDD could be related to cryptogenic liver abscesses.

## Background

PLA has an estimated incidence of 3.6 per 100,000 in the United States but has been reported to be increasing by 4.1% per year [1]. The in hospital mortality of PLA is 5.6% with risk factors being old age, presence of cirrhosis, chronic kidney disease, cancer and septicemia [1]. The majority of PLA cases are associated with biliary tract disease. However, approximately 20–30% cases have no identifiable traditional risk factors and have been labeled as “cryptogenic” [2, 3].

The majority of duodenal diverticula are extraluminal diverticula found in the second part of the duodenum in proximity to the major papilla. The literature describes them as juxta-papillary or peri-ampullary duodenal diverticula. Juxta-papillary duodenal diverticula have been associated with choledocholithiasis, cholelithiasis as well as cholangitis [4]. Although it seems plausible that JPDD may cause pyogenic liver abscesses by promoting infection ascent to the liver, it has not been studied to date. We performed a retrospective case control study to evaluate whether JPDD is associated with cryptogenic PLA.

## Methods

### Study design

This was a retrospective case–control study utilizing the electronic medical record (EMR) database from June 2008 to July 2018 at Elmhurst Hospital, a large teaching community hospital in the New York metropolitan area that services a diverse population. All cases and controls were extracted from the EMR database and reviewed by the first three authors. The first author reviewed all cases of PLA and determined eligibility for inclusion based on prespecified inclusion and exclusion criteria. The same number of age and sex matched controls were identified from the radiology database. The second and third authors reviewed charts of all cases and controls to extract demographic and clinical data. CT scan images were reviewed in the IMPAX software by a single expert radiologist at our institution. Icahn School of Medicine and Elmhurst Hospital institutional board review committees approved the protocol.

### Eligibility criteria for cases and controls

To classify PLA as cases in this study, inclusion criteria included a CT scan diagnosis of liver abscess and either an abscess fluid aspirate positive for bacterial pathogen or resolution of the abscess after empiric antibiotic treatment. We first selected liver abscess cases by using ICD 9 code 572.0. from the discharge diagnosis in the EMR database from July 2008 to June 2018 to identify 89 patients. Then all the resulting charts were reviewed to confirm PLA cases based on our inclusion criteria. The patients with a diagnosis of amoebic liver abscess, traumatic liver abscess, post cholecystectomy liver abscess, concurrent acute cholecystitis, and hepatobiliary malignancy were excluded to focus our study population on cryptogenic PLA cases. Patients with cholelithiasis, choledocholithiasis and cholangitis were not excluded as they have been known to be associated with JPDD [4–6]. After excluding 21 cases who did not meet the study inclusion and exclusion criteria and 2 duplicate charts, a total of 66 cases were included in the current analysis. Using the same radiology database, controls without PLA were identified by one-to-one matching by age, sex and time of initial CT scan of the abdomen and pelvis within 2 weeks of the matched case’s diagnostic scan. 66 matched controls were identified. Thus, data from a total of 132 patients were analyzed and reported for the current study.

### Radiology review to identify JPDD

All the CT scan images were reviewed in the IMPAX software by a single expert radiologist at our institution. The reviewing radiologist confirmed the presence of liver abscess in the included cases and also recorded the number of abscess cavities. The second part of the duodenum was reviewed to record the presence of JPDD.

### Traditional co-morbid risk factors

Abdominal ultrasound and abdominal CT scan results were reviewed to obtain data on bile duct stones and gallstones. Data on the presence of DM was extracted from the patient’s EMR. For the current analysis, the traditional risk factors for PLA included DM, gallstones and bile duct stones. Presence of cholangitis was verified from medical chart reviews with presence of jaundice, fever or leukocytosis and abdominal pain. Cases without traditional risk factors were defined as cryptogenic PLA.

### Demographic and clinical data

In addition to demographic data (age, sex), body mass index (BMI), hypertension and diagnosis of DM were extracted from patient electronic medical records.

### Factors related to liver abscess

For all cases, data on specific bacterial species growth was extracted and recorded as single or poly-microbial growth from blood culture results when available. The number of abscess cavities were recorded from the review of CT scan images.

### Statistical analysis

First, descriptive statistics, counts and frequencies were presented when appropriate. Second, the bivariate associations between risk factors and outcomes were reported by T-test or Chi-square test. For continuous variables including age and BMI, the average age and BMI were compared between case and control groups by using T-test with a significance level of *p* value < 0.05 reported. For the categorical background variables (i.e., sex and hypertension status) and two sets of risk factors including the traditional risk factors – having DM, gallstones, bile duct stones and cholangitis and the study risk factor JPDD Chi-squared tests were applied to investigate whether the distribution of the patients by the diagnosis of PLA differed in these two groups. Third, to assess the magnitude of the relationship between one dependent binary variable and one independent variable, logistic regression was then performed. The dependent variable is whether or not a patient was diagnosed with PLA. An unadjusted and adjusted odds ratio was obtained from the logistic model to describe the likelihood that PLA would occur for each characteristic, with a significance level at *p* value < 0.05 reported. The data analysis for this paper was generated using SAS® software, Version 9.4 of the SAS System for Windows. Copyright © 2021. SAS Institute Inc., Cary, NC, USA.

## Results

Baseline characteristics of patients with PLA (cases, n = 66) and without PLA (controls, n = 66) are presented in Table 1. Mean age at diagnosis was 57.46 for the cases and 57.34 for controls. 82% of patients with PLA were males (n = 54). Comparing cases with controls, there was no statistical difference in the two matching variables, age and sex, as well as BMI and hypertension.

**Table 1:** Background characteristics of the study sample (N = 132)

	Abscess (Case) (N = 66)	No Abscess (Control) (N = 66)	OR (95% CI)	*p *value
	% (N)	% (N)		
Age in years (Mean, SD)	57.5(15.8)	57.4 (15.6)	1.00 (0.98, 1.02)	0.98
Male	81.8 (54)	80.3 (53)	1.10 (0.46, 2.64)	0.82
BMI (Mean, SD)	26.7 (5.7)	26.1 (7.7)	1.01 (0.96, 1.07)	0.63
Hypertension	34.9 (23)	31.8 (21)	1.15 (0.56, 2.37)	0.71

**Fig. 1:**
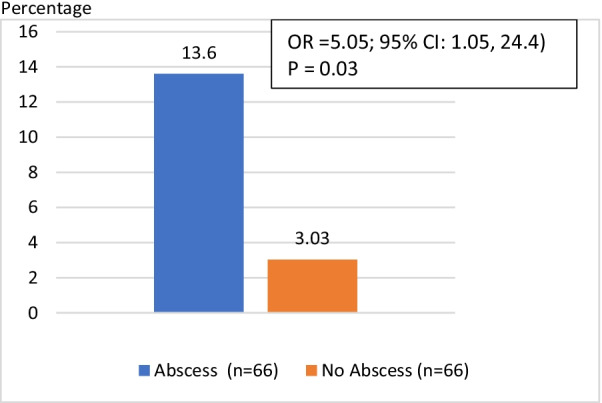
Frequency of JPDD in cases and controls

Table 2 presents two sets of risk factors, the traditional risk factors including DM, gallstones and bile duct stones and the study risk factor JPDD. 13.6% (9/66) of patients with PLA were also found to have JPDD, compared to only 3% (2/66) of patients without PLA (*p* = 0.03). The logistic regression results showed that patients with PLA were 5.05 [OR 5.05; CI 1.05–24.4] times more likely to have a comorbid diagnosis of JPDD than those without PLA (Fig. 1). The result remained statistically significant on multiple logistic regression analysis after controlling for presence of DM and cholangitis. Among five cases with concurrent cholangitis, one had bile duct stone and one had JPDD.

**Table 2:** Risk factors for PLA in cases and controls

Risk factors	Abscess (Case) (N = 66)	No abscess (Control) (N = 66)	OR (95% CI)	*p* value
	% (N)	% (N)		
Traditional risk factors				
DM	42.4 (28)	21.2 (14)	2.74 (1.27, 5.89)	0.01
Gallstones	21.2 (14)	15.6 (10)	1.51 (0.62, 3.69)	0.37
Bile Duct Stones	3.0 (2)	1.5 ( 1)	2.03 (0.18, 23.0)	0.56
Cholangitis	7.6 (5)	0 (0)		NA
Study risk factor				
JPDD	13.6 (9)	3.0 (2)	5.05 (1.05, 24.4)	0.03

Further analysis was performed to explore if the presence of JPDD had association with cryptogenic PLA. On statistical analysis no significant relationship (*p* = 0.33) was found. Although 33% (n = 3/9) of cases with JPDD had no traditional risk factors, 10% (3/32) cases with cryptogenic PLA had JPDD as the only risk factor. However, the number of cases was too small to establish statistical significance (Table 3).

**Table 3:** If JPDD is the only risk factor for patients with PLA (n = 66)

	Cryptogenic PLA				*p* value
	Yes N (%)		No N (%)		
Non-JPDD	29	(50.9)	28	(49.1)	0.33
JPDD	3	(33.3)	6	(66.7)	

We also found that about 42% (n = 28/66) of cases had a diagnosis of DM compared to only 21% (n = 14/66) of controls. This association was statistically significant (*p* = 0.01) so further analysis was performed to explore relation of DM to other comorbid conditions. Table 4 shows that significantly more patients (35.7%) with comorbid DM and liver abscesses had gall stones compared to PLA patients without DM (*p* = 0.04). No other conditions showed such a relationship.

**Table 4:** DM and other comorbid conditions by PLA status

	Diabetic		Not diabetic		*p *value
	PLA (N = 28)	No PLA (N = 14)	PLA (N = 38)	No PLA (N = 52)	
	% (N)	% (N)	% (N)	% (N)	
Gallstones	35.7 (10)	21.4 (3)	10.5 (4)	13.5 (7)	0.04
Bile Duct Stones	3.6 (1)	0 (0)	2.6 (1)	1.9 (1)	0.90
JPDD	10.7 (3)	7.1 (1)	15.8 (6)	1.9 (1)	0.12

## Discussion

This is the first time a relation between the presence of JPDD and PLA has been reported. We found that patients with PLA have OR of 5.05 to have a comorbid diagnosis of JPDD. Consistent with previously published results, presence of DM was also found to have statistically significant association with PLA.

There is previously reported data showing association of JPDD with biliary tract disease [4–6] which provides biological plausibility to support our result. JPDD are defined as extraluminal diverticula of the duodenum located adjacent to the ampulla of Vater. JPDD have variable incidence rates ranging from 4.1 to 50% which are highly dependent on the modality of diagnosis and cohort age group. However, the majority of studies show that the incidence of JPDD increases with age with rate reported as high as 45% among patients with age above 70 [7, 8]. Past studies show CT scan is highly sensitive in diagnosing JPDD [9].

Presence of JPDD has been shown to affect function of the sphincter of Oddi. Intraoperative cholangiography in patients with JPDD who had cholecystectomy demonstrated mechanical obstruction of the terminal biliary ductal system in the presence of a duodenal pressure load [10]. Patients with JPDD have been shown to have significantly higher bacterial overgrowth in the duodenum compared to those without diverticula. Skar et al. found that the most common bacteria isolated from the duodenum were *Escherichia coli*, *Klebsiella* sp, and *Streptococcus faecalis* which are the same bacteria commonly associated with PLA [11]. These alterations lead to increased bile stasis which in turn may result in increased incidence of bile duct stones as well as infectious complications. Several studies including a recent meta-analysis have shown increased incidence of bile duct stones in patients with JPDD [5, 12]. JPDD have been associated with obstructive jaundice in the absence of choledocholithiasis, described as Lemmel’s syndrome [13]. This syndrome has also been described in patient presenting with cholangitis [14]. In our study, five (7.6%) cases with PLA had concurrent cholangitis. One of them had JPDD but no bile duct stones suggesting Lemmel’s syndrome. Interestingly, our results suggest JPDD remains a risk for PLA in the absence of concurrent cholangitis.

In the published literature the majority of PLAs are described as being secondary to biliary diseases [1, 3]. Furthermore, the number of published cryptogenic PLA has been increasing [2, 15]. We found that about 10% of patients with cryptogenic PLA in our study had JPDD and one third of the patients with PLA having JPDD did not have any other traditional risk factors. Although in this small group there was no statistically significant association of JPDD as a sole risk factor for cryptogenic PLA, our results may suggest a possible etiological role for JPDD’s in cryptogenic PLA.

Our study findings of a higher rate of DM in the patients with PLA, higher proportion of males among the cases as well as positive association of gall stones with presence of DM are consistent with previous studies [16–18]. An expert radiologist reviewed the CT scan images to extract data on presence of JPDD and confirmed the presence of PLA and its characteristics. CT scan is highly sensitive for diagnosis of JPDD. Our study does show an association but did not establish causality. Limitations of our study include its retrospective nature as well as the fact that we did not have a large enough number of patients with cryptogenic PLA to show a statistically significant association with JPDD.

## Conclusion

In conclusion, our study is the first to demonstrate an association of JPDD with PLA. However, our study did not have sample size large enough to establish an association between JPDD and cryptogenic PLA. Larger studies are needed to validate our findings, and to evaluate the etiological relationship between JPDD and cryptogenic PLA.

## Data Availability

The de-identified data, analytical methods and study materials will be made available for other researchers upon request.

## Abbreviations

JPDD: Juxta-papillary duodenal diverticulum
PLA: Pyogenic liver abscess
ICD: International classification of diseases
EMR: Electronic medical records
CT: Computerized tomography
OR: Odds ratio
DM: Diabetes mellitus
BMI: Body mass index
